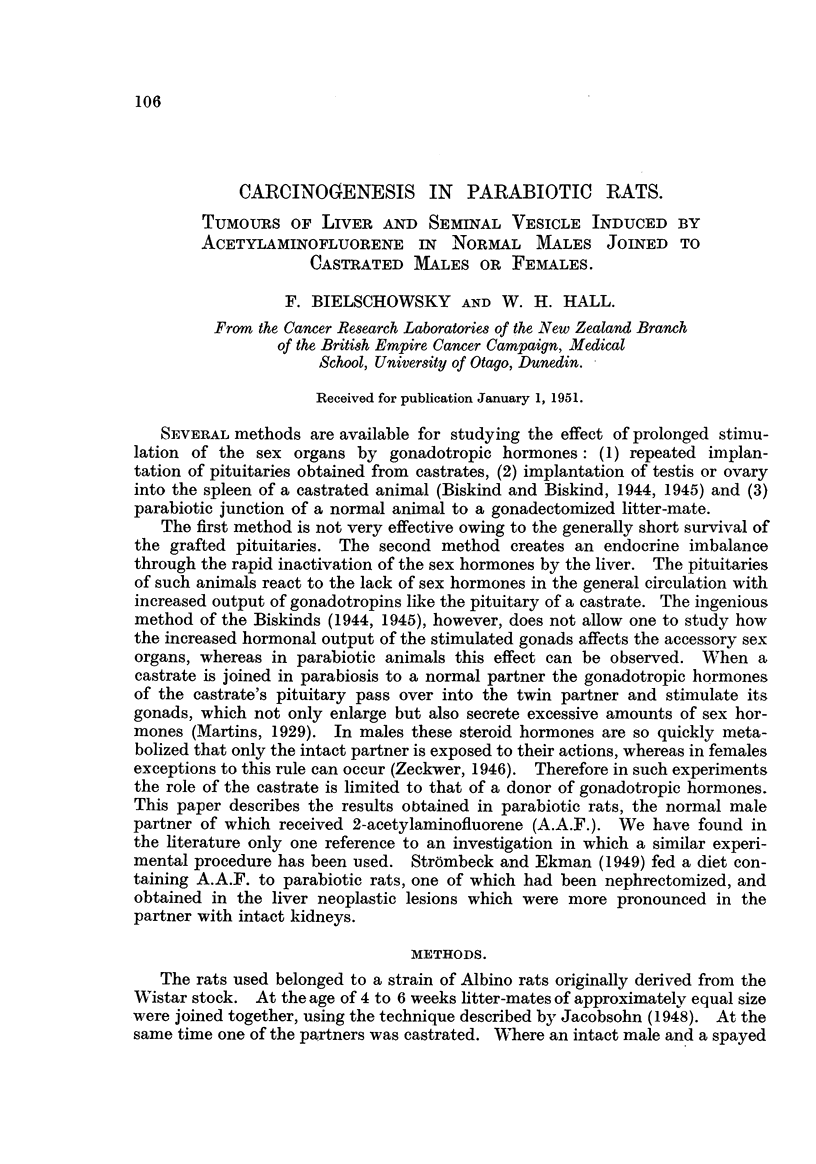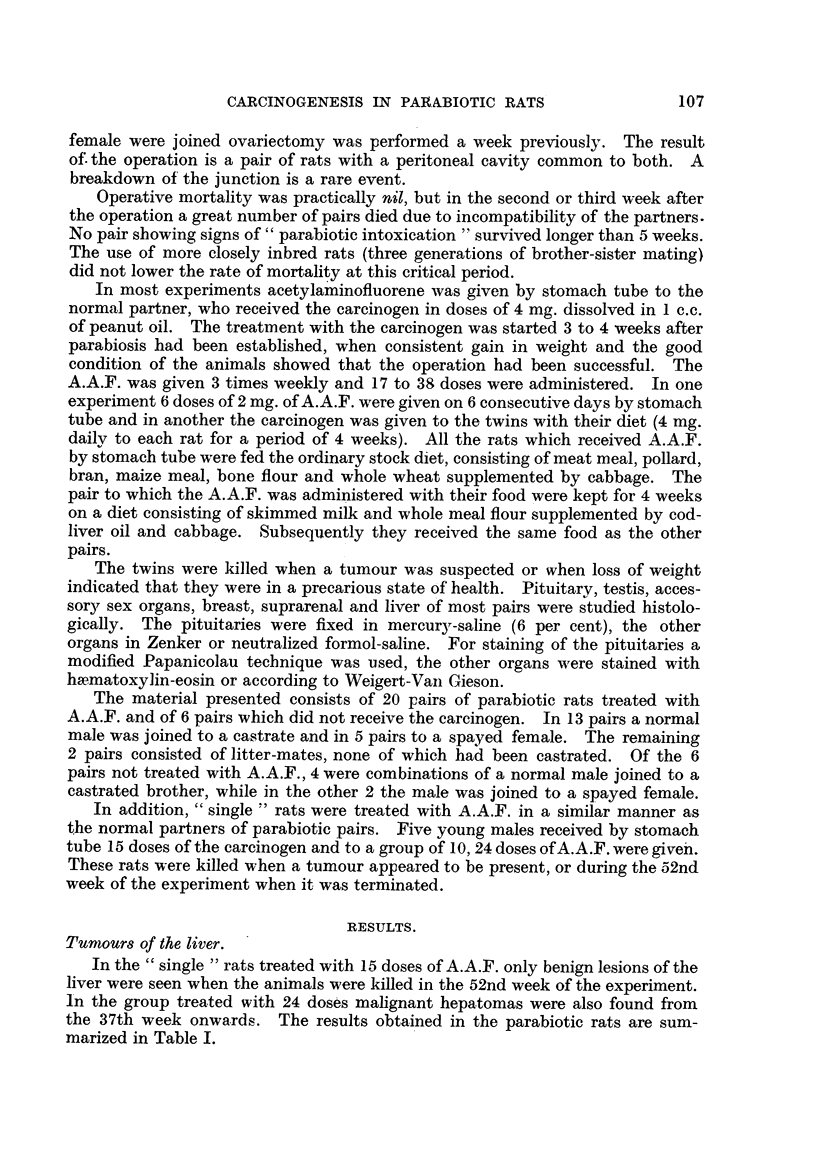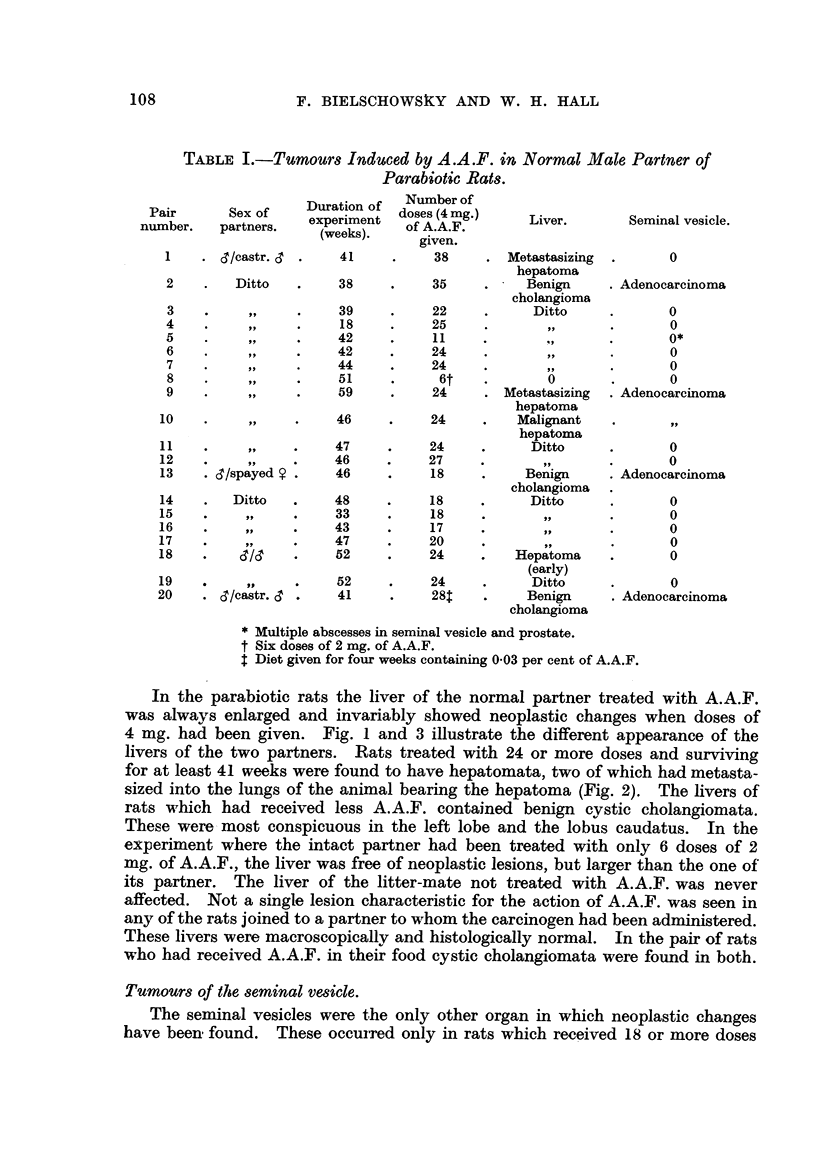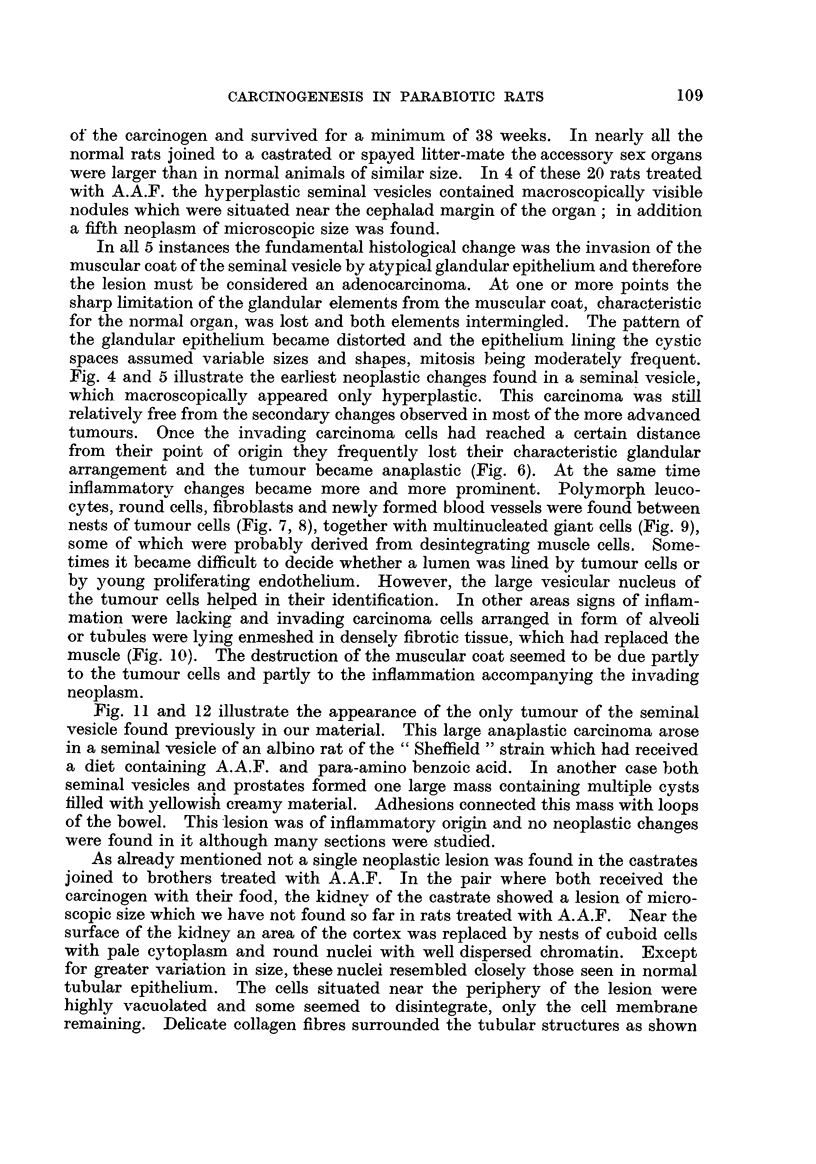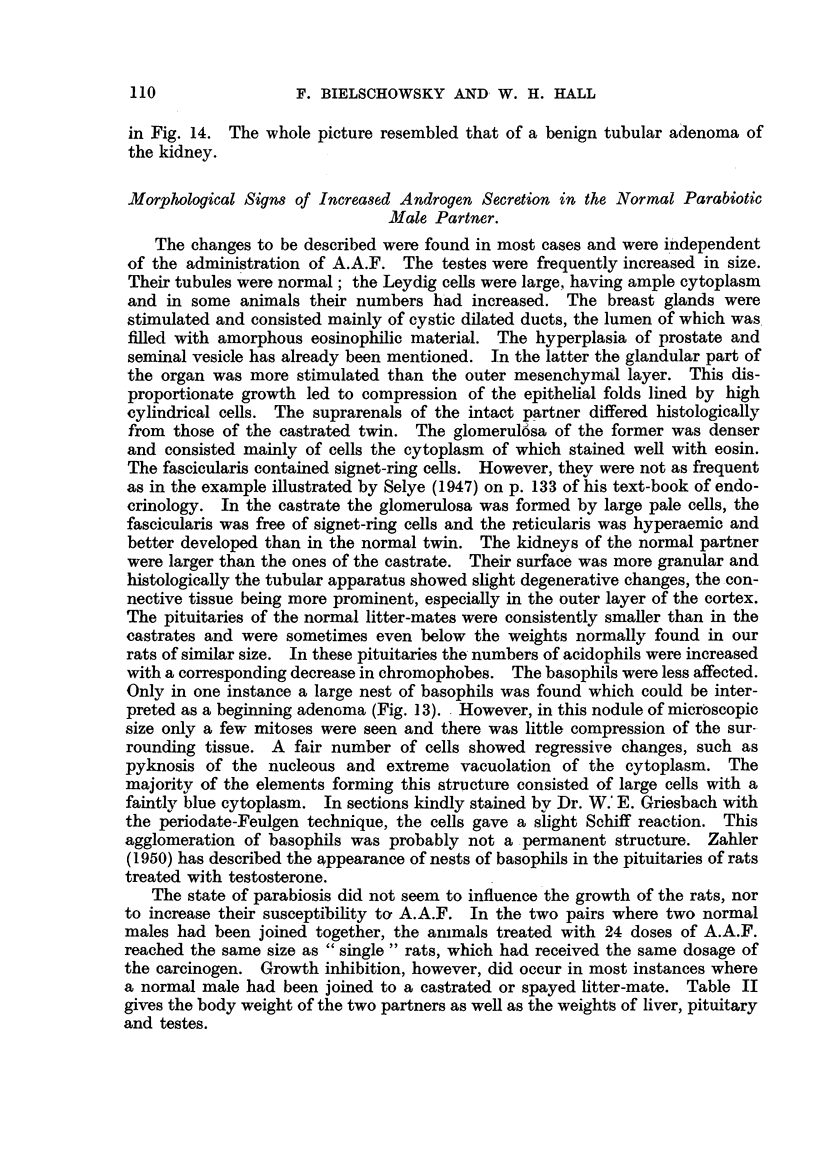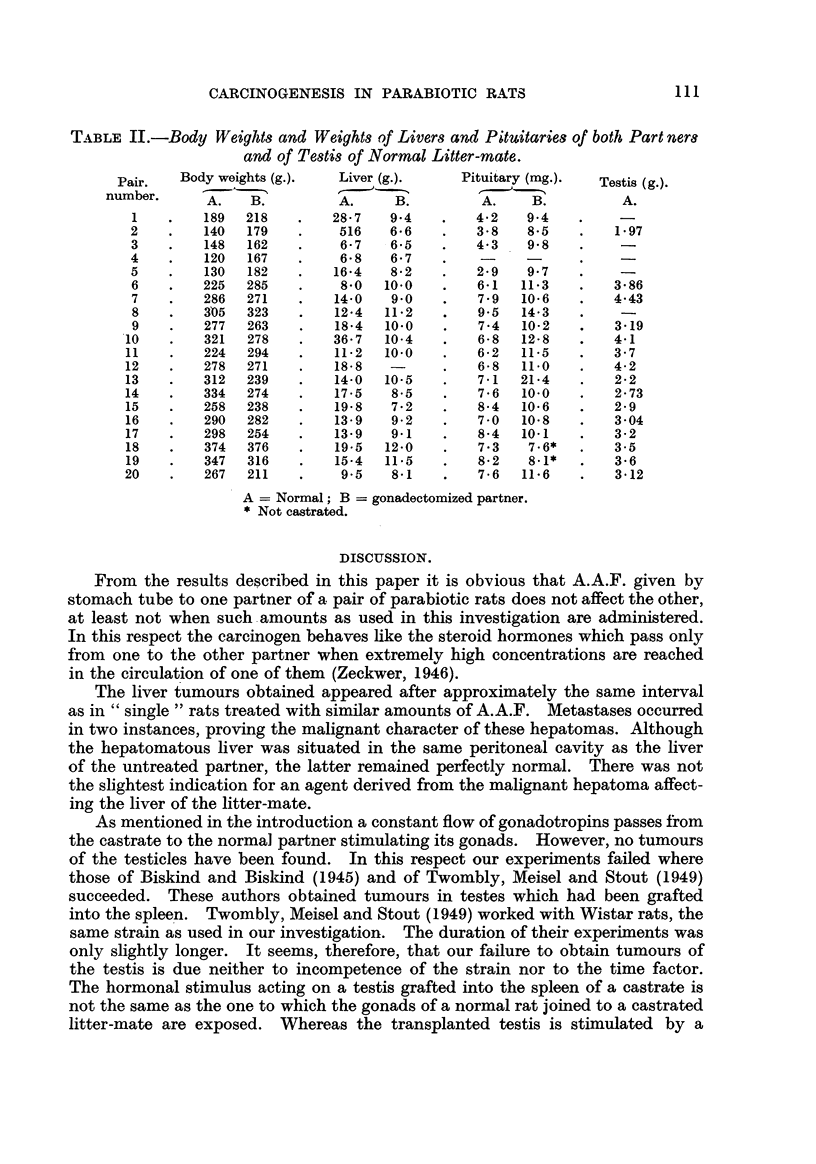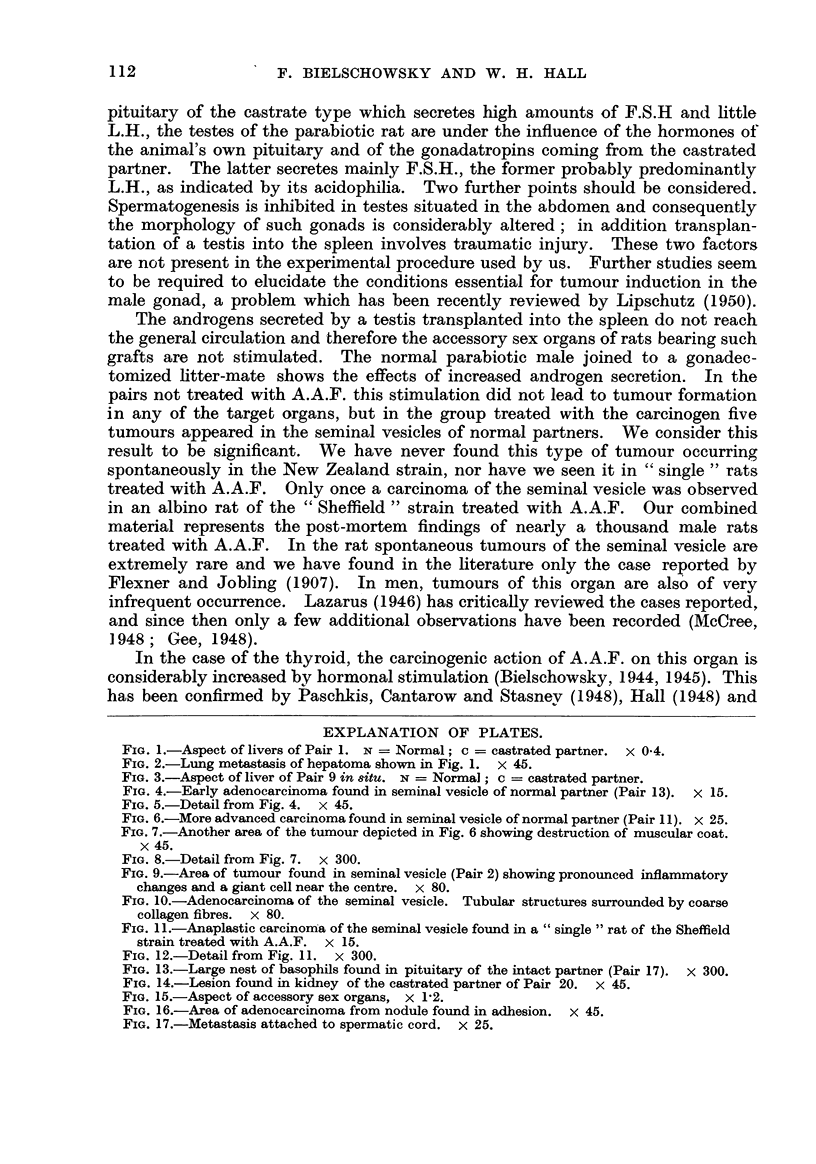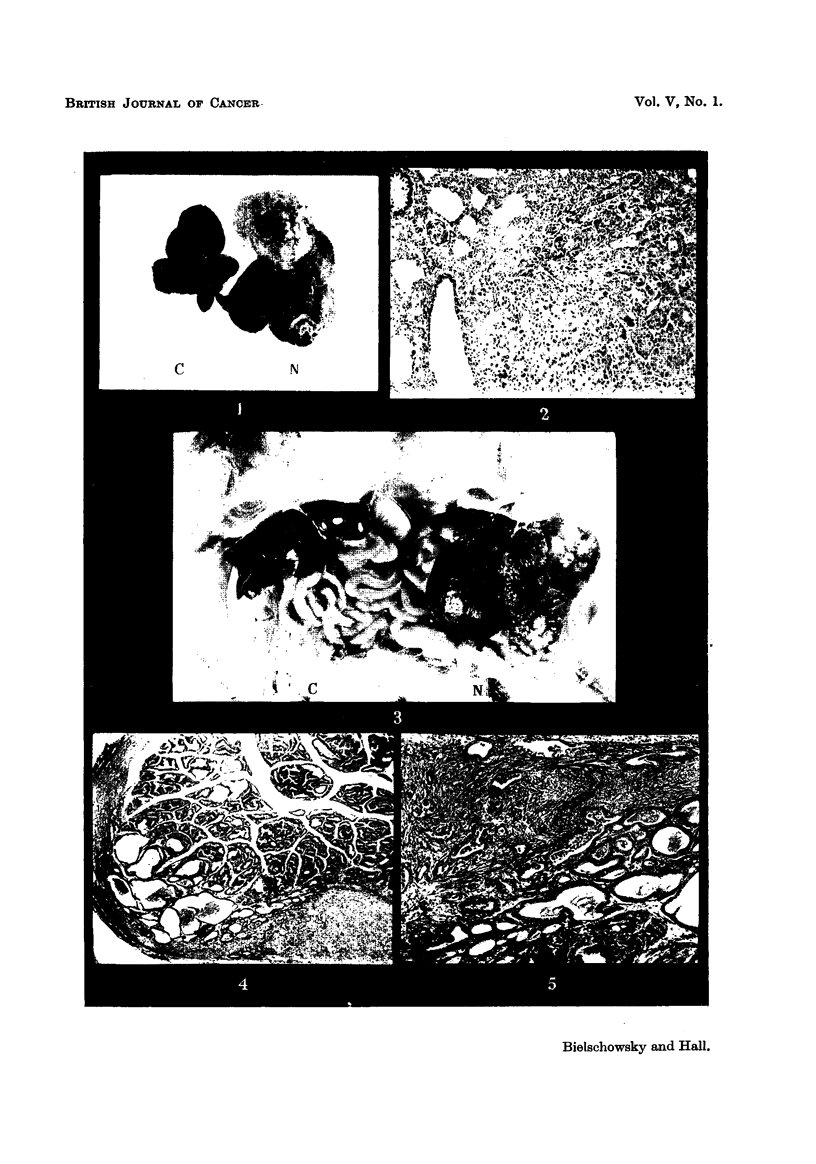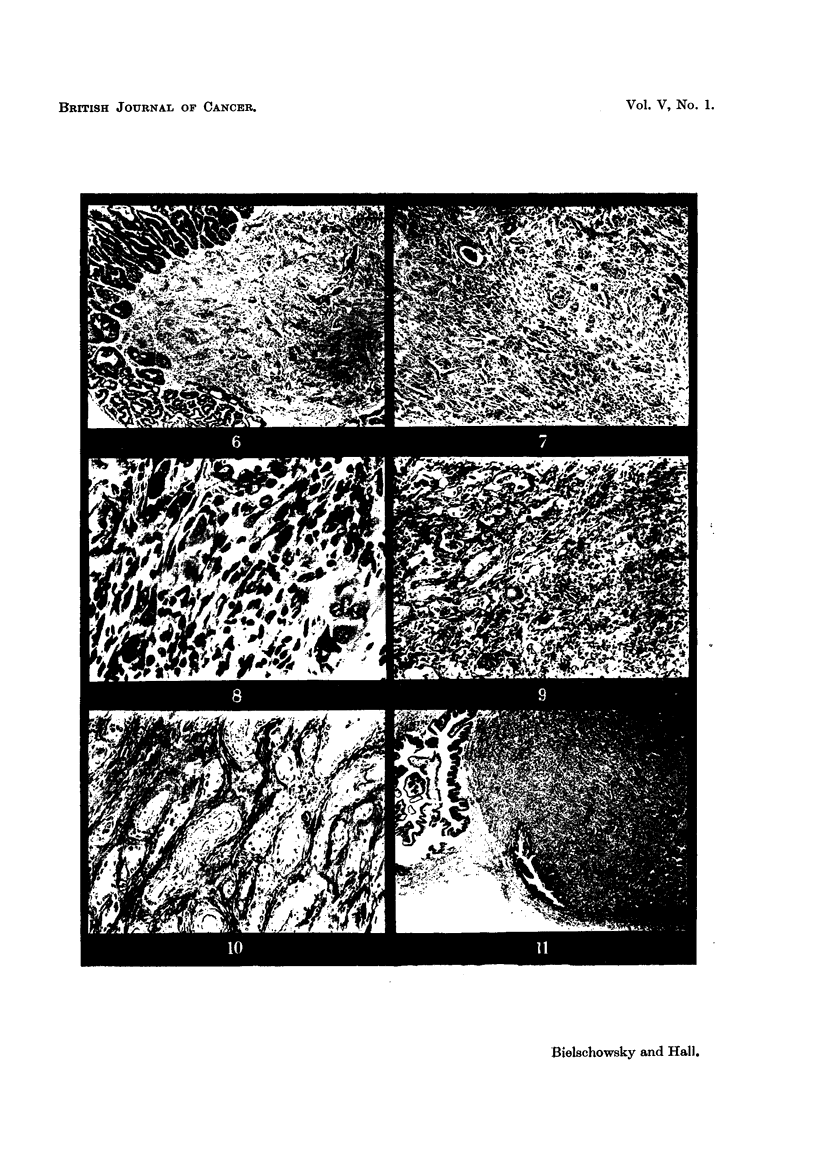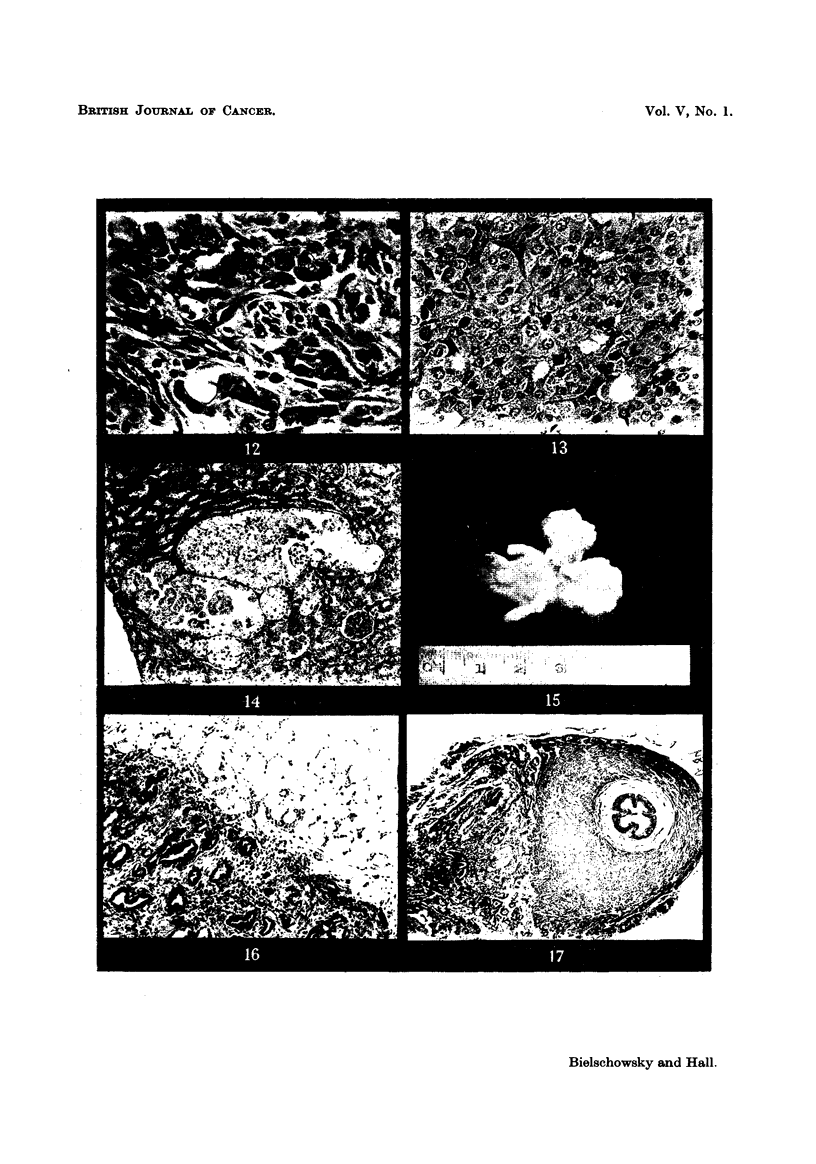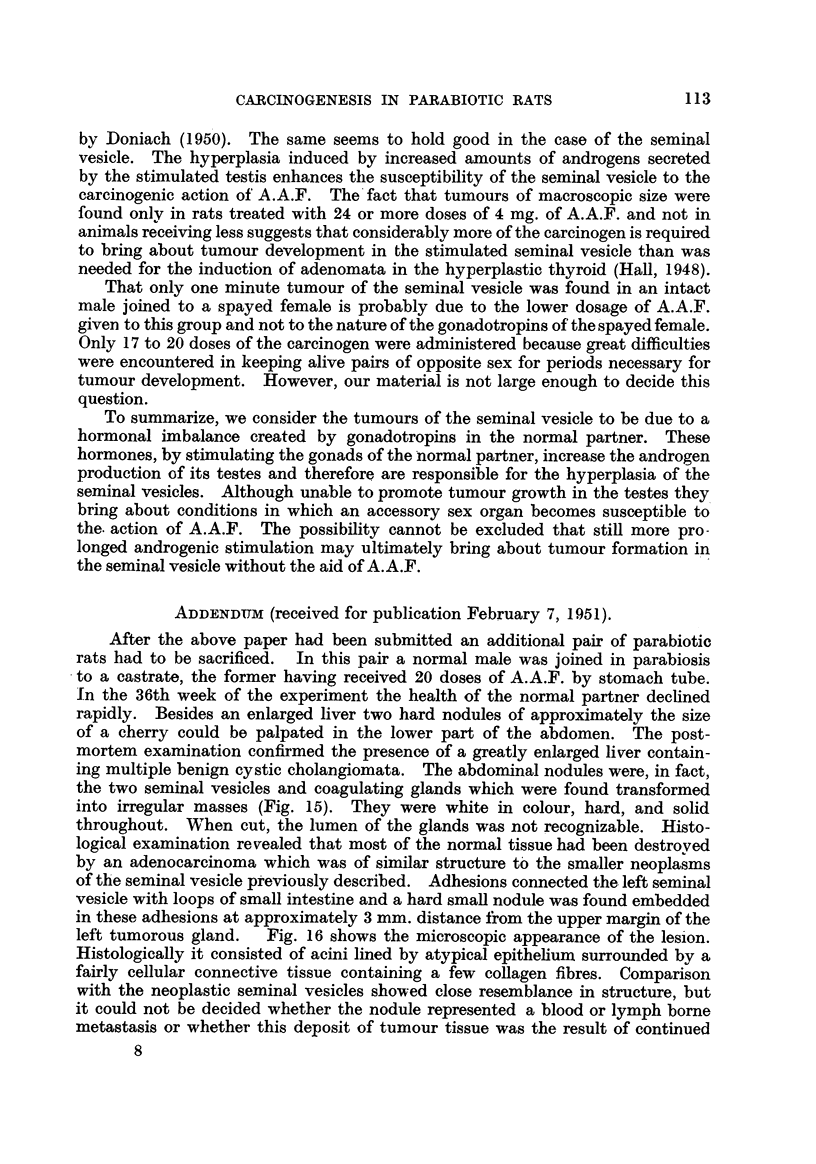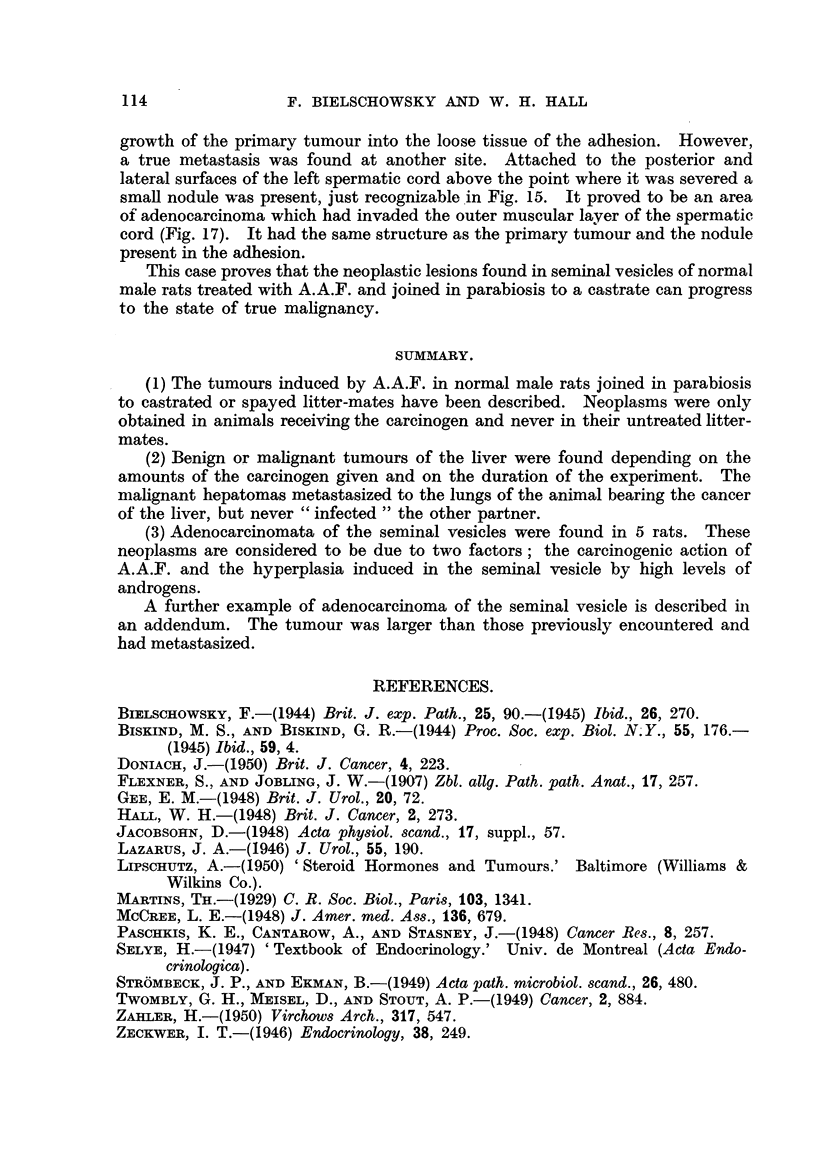# Carcinogenesis in Parabiotic Rats

**DOI:** 10.1038/bjc.1951.10

**Published:** 1951-03

**Authors:** F. Bielschowsky, W. H. Hall

## Abstract

**Images:**


### CARCINOGENESIS IN PARABIOTIC RATS.

### Tumours of Liver and Seminal Vesicle Induced by Acetylaminofluorene in Normal Males Joined to Castrated Males or Females.

F. BIELSCHOWSKY and W. H. HALL.

*From the Cancer Research Laboratories of the New Zealand Branch of the British Empire Cancer Campaign, Medical School, University of Otago, Dunedin.*

Received for publication January 1, 1951.

Several methods are available for studying the effect of prolonged stimulation of the sex organs by gonadotropic hormones: (1) repeated implantation of pituitaries obtained from castrates, (2) implantation of testis or ovary into the spleen of a castrated animal (Biskind and Biskind, 1944, 1945) and (3) parabiotic junction of a normal animal to a gonadectomized litter-mate.

The first method is not very effective owing to the generally short survival of the grafted pituitaries. The second method creates an endocrine imbalance through the rapid inactivation of the sex hormones by the liver. The pituitaries of such animals react to the lack of sex hormones in the general circulation with increased output of gonadotropins like the pituitary of a castrate. The ingenious method of the Biskinds (1944, 1945), however, does not allow one to study how the increased hormonal output of the stimulated gonads affects the accessory sex organs, whereas in parabiotic animals this effect can be observed. When a castrate is joined in parabiosis to a normal partner the gonadotropic hormones of the castrate's pituitary pass over into the twin partner and stimulate its gonads, which not only enlarge but also secrete excessive amounts of sex hormones (Martins, 1929). In males these steroid hormones are so quickly metabolized that only the intact partner is exposed to their actions, whereas in females exceptions to this rule can occur (Zeckwer, 1946). Therefore in such experiments the role of the castrate is limited to that of a donor of gonadotropic hormones. This paper describes the results obtained in parabiotic rats, the normal male partner of which received 2-acetylaminofluorene (A.A.F.). We have found in the literature only one reference to an investigation in which a similar experimental procedure has been used. Strömbeck and Ekman (1949) fed a diet containing A.A.F. to parabiotic rats, one of which had been nephrectomized, and obtained in the liver neoplastic lesions which were more pronounced in the partner with intact kidneys.

### METHODS.

The rats used belonged to a strain of Albino rats originally derived from the Wistar stock. At the age of 4 to 6 weeks litter-mates of approximately equal size were joined together, using the technique described by Jacobsohn (1948). At the same time one of the partners was castrated. Where an intact male and a spayed female were joined ovariectomy was performed a week previously. The result of the operation is a pair of rats with a peritoneal cavity common to both. A breakdown of the junction is a rare event.

Operative mortality was practically *nil*, but in the second or third week after the operation a great number of pairs died due to incompatibility of the partners. No pair showing signs of " parabiotic intoxication " survived longer than 5 weeks. The use of more closely inbred rats (three generations of brother-sister mating) did not lower the rate of mortality at this critical period.

In most experiments acetylaminofluorene was given by stomach tube to the normal partner, who received the carcinogen in doses of 4 mg. dissolved in 1 c.c. of peanut oil. The treatment with the carcinogen was started 3 to 4 weeks after parabiosis had been established, when consistent gain in weight and the good condition of the animals showed that the operation had been successful. The A.A.F. was given 3 times weekly and 17 to 38 doses were administered. In one experiment 6 doses of 2 mg. of A.A.F. were given on 6 consecutive days by stomach tube and in another the carcinogen was given to the twins with their diet (4 mg. daily to each rat for a period of 4 weeks). All the rats which received A.A.F. by stomach tube were fed the ordinary stock diet, consisting of meat meal, pollard, bran, maize meal, bone flour and whole wheat supplemented by cabbage. The pair to which the A.A.F. was administered with their food were kept for 4 weeks on a diet consisting of skimmed milk and whole meal flour supplemented by cod-liver oil and cabbage. Subsequently they received the same food as the other pairs.

The twins were killed when a tumour was suspected or when loss of weight indicated that they were in a precarious state of health. Pituitary, testis, accessory sex organs, breast, suprarenal and liver of most pairs were studied histologically. The pituitaries were fixed in mercury-saline (6 per cent), the other organs in Zenker or neutralized formol-saline. For staining of the pituitaries a modified Papanicolau technique was used, the other organs were stained with hæmatoxylin-eosin or according to Weigert-Van Gieson.

The material presented consists of 20 pairs of parabiotic rats treated with A.A.F. and of 6 pairs which did not receive the carcinogen. In 13 pairs a normal male was joined to a castrate and in 5 pairs to a spayed female. The remaining 2 pairs consisted of litter-mates, none of which had been castrated. Of the 6 pairs not treated with A.A.F., 4 were combinations of a normal male joined to a castrated brother, while in the other 2 the male was joined to a spayed female.

In addition, " single " rats were treated with A.A.F. in a similar manner as the normal partners of parabiotic pairs. Five young males received by stomach tube 15 doses of the carcinogen and to a group of 10, 24 doses of A.A.F. were given. These rats were killed when a tumour appeared to be present, or during the 52nd week of the experiment when it was terminated.

### RESULTS.

*Tumours of the liver.*

In the " single " rats treated with 15 doses of A.A.F. only benign lesions of the liver were seen when the animals were killed in the 52nd week of the experiment. In the group treated with 24 doses malignant hepatomas were also found from the 37th week onwards. The results obtained in the parabiotic rats are summarized in Table I.

TABLE I.—*Tumours Induced by A.A.F. in Normal Male Partner of Parabiotic Rats.*

| Pair number. | Sex of partners. | Duration of experiment (weeks). | Number of doses (4 mg.) of A.A.F. given. | Liver. | Seminal vesicle. |
|---|---|---|---|---|---|
| 1 | ♂/castr. ♂ | 41 | 38 | Metastasizing hepatoma | 0 |
| 2 | Ditto | 38 | 35 | Benign cholangioma | Adenocarcinoma |
| 3 | ,, | 39 | 22 | Ditto | 0 |
| 4 | ,, | 18 | 25 | ,, | 0 |
| 5 | ,, | 42 | 11 | ,, | 0* |
| 6 | ,, | 42 | 24 | ,, | 0 |
| 7 | ,, | 44 | 24 | ,, | 0 |
| 8 | ,, | 51 | 6† | 0 | 0 |
| 9 | ,, | 59 | 24 | Metastasizing hepatoma | Adenocarcinoma |
| 10 | ,, | 46 | 24 | Malignant hepatoma | ,, |
| 11 | ,, | 47 | 24 | Ditto | 0 |
| 12 | ,, | 46 | 27 | ,, | 0 |
| 13 | ♂/spayed ♀ | 46 | 18 | Benign cholangioma | Adenocarcinoma |
| 14 | Ditto | 48 | 18 | Ditto | 0 |
| 15 | ,, | 33 | 18 | ,, | 0 |
| 16 | ,, | 43 | 17 | ,, | 0 |
| 17 | ,, | 47 | 20 | ,, | 0 |
| 18 | ♂/♂ | 52 | 24 | Hepatoma (early) | 0 |
| 19 | ,, | 52 | 24 | Ditto | 0 |
| 20 | ♂/castr. ♂ | 41 | 28‡ | Benign cholangioma | Adenocarcinoma |

* Multiple abscesses in seminal vesicle and prostate.
† Six doses of 2 mg. of A.A.F.
‡ Diet given for four weeks containing 0·03 per cent of A.A.F.

In the parabiotic rats the liver of the normal partner treated with A.A.F. was always enlarged and invariably showed neoplastic changes when doses of 4 mg. had been given. Fig. 1 and 3 illustrate the different appearance of the livers of the two partners. Rats treated with 24 or more doses and surviving for at least 41 weeks were found to have hepatomata, two of which had metastasized into the lungs of the animal bearing the hepatoma (Fig. 2). The livers of rats which had received less A.A.F. contained benign cystic cholangiomata. These were most conspicuous in the left lobe and the lobus caudatus. In the experiment where the intact partner had been treated with only 6 doses of 2 mg. of A.A.F., the liver was free of neoplastic lesions, but larger than the one of its partner. The liver of the litter-mate not treated with A.A.F. was never affected. Not a single lesion characteristic for the action of A.A.F. was seen in any of the rats joined to a partner to whom the carcinogen had been administered. These livers were macroscopically and histologically normal. In the pair of rats who had received A.A.F. in their food cystic cholangiomata were found in both.

*Tumours of the seminal vesicle.*

The seminal vesicles were the only other organ in which neoplastic changes have been found. These occurred only in rats which received 18 or more doses of the carcinogen and survived for a minimum of 38 weeks. In nearly all the normal rats joined to a castrated or spayed litter-mate the accessory sex organs were larger than in normal animals of similar size. In 4 of these 20 rats treated with A.A.F. the hyperplastic seminal vesicles contained macroscopically visible nodules which were situated near the cephalad margin of the organ ; in addition a fifth neoplasm of microscopic size was found.

In all 5 instances the fundamental histological change was the invasion of the muscular coat of the seminal vesicle by atypical glandular epithelium and therefore the lesion must be considered an adenocarcinoma. At one or more points the sharp limitation of the glandular elements from the muscular coat, characteristic for the normal organ, was lost and both elements intermingled. The pattern of the glandular epithelium became distorted and the epithelium lining the cystic spaces assumed variable sizes and shapes, mitosis being moderately frequent. Fig. 4 and 5 illustrate the earliest neoplastic changes found in a seminal vesicle, which macroscopically appeared only hyperplastic. This carcinoma was still relatively free from the secondary changes observed in most of the more advanced tumours. Once the invading carcinoma cells had reached a certain distance from their point of origin they frequently lost their characteristic glandular arrangement and the tumour became anaplastic (Fig. 6). At the same time inflammatory changes became more and more prominent. Polymorph leucocytes, round cells, fibroblasts and newly formed blood vessels were found between nests of tumour cells (Fig. 7, 8), together with multinucleated giant cells (Fig. 9), some of which were probably derived from desintegrating muscle cells. Sometimes it became difficult to decide whether a lumen was lined by tumour cells or by young proliferating endothelium. However, the large vesicular nucleus of the tumour cells helped in their identification. In other areas signs of inflammation were lacking and invading carcinoma cells arranged in form of alveoli or tubules were lying enmeshed in densely fibrotic tissue, which had replaced the muscle (Fig. 10). The destruction of the muscular coat seemed to be due partly to the tumour cells and partly to the inflammation accompanying the invading neoplasm.

Fig. 11 and 12 illustrate the appearance of the only tumour of the seminal vesicle found previously in our material. This large anaplastic carcinoma arose in a seminal vesicle of an albino rat of the " Sheffield " strain which had received a diet containing A.A.F. and para-amino benzoic acid. In another case both seminal vesicles and prostates formed one large mass containing multiple cysts filled with yellowish creamy material. Adhesions connected this mass with loops of the bowel. This lesion was of inflammatory origin and no neoplastic changes were found in it although many sections were studied.

As already mentioned not a single neoplastic lesion was found in the castrates joined to brothers treated with A.A.F. In the pair where both received the carcinogen with their food, the kidney of the castrate showed a lesion of microscopic size which we have not found so far in rats treated with A.A.F. Near the surface of the kidney an area of the cortex was replaced by nests of cuboid cells with pale cytoplasm and round nuclei with well dispersed chromatin. Except for greater variation in size, these nuclei resembled closely those seen in normal tubular epithelium. The cells situated near the periphery of the lesion were highly vacuolated and some seemed to disintegrate, only the cell membrane remaining. Delicate collagen fibres surrounded the tubular structures as shown in Fig. 14. The whole picture resembled that of a benign tubular adenoma of the kidney.

*Morphological Signs of Increased Androgen Secretion in the Normal Parabiotic Male Partner.*

The changes to be described were found in most cases and were independent of the administration of A.A.F. The testes were frequently increased in size. Their tubules were normal; the Leydig cells were large, having ample cytoplasm and in some animals their numbers had increased. The breast glands were stimulated and consisted mainly of cystic dilated ducts, the lumen of which was filled with amorphous eosinophilic material. The hyperplasia of prostate and seminal vesicle has already been mentioned. In the latter the glandular part of the organ was more stimulated than the outer mesenchymal layer. This disproportionate growth led to compression of the epithelial folds lined by high cylindrical cells. The suprarenals of the intact partner differed histologically from those of the castrated twin. The glomerulosa of the former was denser and consisted mainly of cells the cytoplasm of which stained well with eosin. The fascicularis contained signet-ring cells. However, they were not as frequent as in the example illustrated by Selye (1947) on p. 133 of his text-book of endocrinology. In the castrate the glomerulosa was formed by large pale cells, the fascicularis was free of signet-ring cells and the reticularis was hyperaemic and better developed than in the normal twin. The kidneys of the normal partner were larger than the ones of the castrate. Their surface was more granular and histologically the tubular apparatus showed slight degenerative changes, the connective tissue being more prominent, especially in the outer layer of the cortex. The pituitaries of the normal litter-mates were consistently smaller than in the castrates and were sometimes even below the weights normally found in our rats of similar size. In these pituitaries the numbers of acidophils were increased with a corresponding decrease in chromophobes. The basophils were less affected. Only in one instance a large nest of basophils was found which could be interpreted as a beginning adenoma (Fig. 13). However, in this nodule of microscopic size only a few mitoses were seen and there was little compression of the surrounding tissue. A fair number of cells showed regressive changes, such as pyknosis of the nucleous and extreme vacuolation of the cytoplasm. The majority of the elements forming this structure consisted of large cells with a faintly blue cytoplasm. In sections kindly stained by Dr. W. E. Griesbach with the periodate-Feulgen technique, the cells gave a slight Schiff reaction. This agglomeration of basophils was probably not a permanent structure. Zahler (1950) has described the appearance of nests of basophils in the pituitaries of rats treated with testosterone.

The state of parabiosis did not seem to influence the growth of the rats, nor to increase their susceptibility to A.A.F. In the two pairs where two normal males had been joined together, the animals treated with 24 doses of A.A.F. reached the same size as " single " rats, which had received the same dosage of the carcinogen. Growth inhibition, however, did occur in most instances where a normal male had been joined to a castrated or spayed litter-mate. Table II gives the body weight of the two partners as well as the weights of liver, pituitary and testes.

TABLE II.—*Body Weights and Weights of Livers and Pituitaries of both Partners and of Testis of Normal Litter-mate.*

| Pair number. | Body weights (g.). A. | B. | Liver (g.). A. | B. | Pituitary (mg.). A. | B. | Testis (g.). A. |
|---|---|---|---|---|---|---|---|
| 1 | 189 | 218 | 28·7 | 9·4 | 4·2 | 9·4 | — |
| 2 | 140 | 179 | 516 | 6·6 | 3·8 | 8·5 | 1·97 |
| 3 | 148 | 162 | 6·7 | 6·5 | 4·3 | 9·8 | — |
| 4 | 120 | 167 | 6·8 | 6·7 | — | — | — |
| 5 | 130 | 182 | 16·4 | 8·2 | 2·9 | 9·7 | — |
| 6 | 225 | 285 | 8·0 | 10·0 | 6·1 | 11·3 | 3·86 |
| 7 | 286 | 271 | 14·0 | 9·0 | 7·9 | 10·6 | 4·43 |
| 8 | 305 | 323 | 12·4 | 11·2 | 9·5 | 14·3 | — |
| 9 | 277 | 263 | 18·4 | 10·0 | 7·4 | 10·2 | 3·19 |
| 10 | 321 | 278 | 36·7 | 10·4 | 6·8 | 12·8 | 4·1 |
| 11 | 224 | 294 | 11·2 | 10·0 | 6·2 | 11·5 | 3·7 |
| 12 | 278 | 271 | 18·8 | — | 6·8 | 11·0 | 4·2 |
| 13 | 312 | 239 | 14·0 | 10·5 | 7·1 | 21·4 | 2·2 |
| 14 | 334 | 274 | 17·5 | 8·5 | 7·6 | 10·0 | 2·73 |
| 15 | 258 | 238 | 19·8 | 7·2 | 8·4 | 10·6 | 2·9 |
| 16 | 290 | 282 | 13·9 | 9·2 | 7·0 | 10·8 | 3·04 |
| 17 | 298 | 254 | 13·9 | 9·1 | 8·4 | 10·1 | 3·2 |
| 18 | 374 | 376 | 19·5 | 12·0 | 7·3 | 7·6* | 3·5 |
| 19 | 347 | 316 | 15·4 | 11·5 | 8·2 | 8·1* | 3·6 |
| 20 | 267 | 211 | 9·5 | 8·1 | 7·6 | 11·6 | 3·12 |

A = Normal; B = gonadectomized partner.
* Not castrated.

### DISCUSSION.

From the results described in this paper it is obvious that A.A.F. given by stomach tube to one partner of a pair of parabiotic rats does not affect the other, at least not when such amounts as used in this investigation are administered. In this respect the carcinogen behaves like the steroid hormones which pass only from one to the other partner when extremely high concentrations are reached in the circulation of one of them (Zeckwer, 1946).

The liver tumours obtained appeared after approximately the same interval as in " single " rats treated with similar amounts of A.A.F. Metastases occurred in two instances, proving the malignant character of these hepatomas. Although the hepatomatous liver was situated in the same peritoneal cavity as the liver of the untreated partner, the latter remained perfectly normal. There was not the slightest indication for an agent derived from the malignant hepatoma affecting the liver of the litter-mate.

As mentioned in the introduction a constant flow of gonadotropins passes from the castrate to the normal partner stimulating its gonads. However, no tumours of the testicles have been found. In this respect our experiments failed where those of Biskind and Biskind (1945) and of Twombly, Meisel and Stout (1949) succeeded. These authors obtained tumours in testes which had been grafted into the spleen. Twombly, Meisel and Stout (1949) worked with Wistar rats, the same strain as used in our investigation. The duration of their experiments was only slightly longer. It seems, therefore, that our failure to obtain tumours of the testis is due neither to incompetence of the strain nor to the time factor. The hormonal stimulus acting on a testis grafted into the spleen of a castrate is not the same as the one to which the gonads of a normal rat joined to a castrated litter-mate are exposed. Whereas the transplanted testis is stimulated by a pituitary of the castrate type which secretes high amounts of F.S.H and little L.H., the testes of the parabiotic rat are under the influence of the hormones of the animal's own pituitary and of the gonadatropins coming from the castrated partner. The latter secretes mainly F.S.H., the former probably predominantly L.H., as indicated by its acidophilia. Two further points should be considered. Spermatogenesis is inhibited in testes situated in the abdomen and consequently the morphology of such gonads is considerably altered ; in addition transplantation of a testis into the spleen involves traumatic injury. These two factors are not present in the experimental procedure used by us. Further studies seem to be required to elucidate the conditions essential for tumour induction in the male gonad, a problem which has been recently reviewed by Lipschutz (1950).

The androgens secreted by a testis transplanted into the spleen do not reach the general circulation and therefore the accessory sex organs of rats bearing such grafts are not stimulated. The normal parabiotic male joined to a gonadectomized litter-mate shows the effects of increased androgen secretion. In the pairs not treated with A.A.F. this stimulation did not lead to tumour formation in any of the target organs, but in the group treated with the carcinogen five tumours appeared in the seminal vesicles of normal partners. We consider this result to be significant. We have never found this type of tumour occurring spontaneously in the New Zealand strain, nor have we seen it in " single " rats treated with A.A.F. Only once a carcinoma of the seminal vesicle was observed in an albino rat of the " Sheffield " strain treated with A.A.F. Our combined material represents the post-mortem findings of nearly a thousand male rats treated with A.A.F. In the rat spontaneous tumours of the seminal vesicle are extremely rare and we have found in the literature only the case reported by Flexner and Jobling (1907). In men, tumours of this organ are also of very infrequent occurrence. Lazarus (1946) has critically reviewed the cases reported, and since then only a few additional observations have been recorded (McCree, 1948 ; Gee, 1948).

In the case of the thyroid, the carcinogenic action of A.A.F. on this organ is considerably increased by hormonal stimulation (Bielschowsky, 1944, 1945). This has been confirmed by Paschkis, Cantarow and Stasney (1948), Hall (1948) and

EXPLANATION OF PLATES.

Fig. 1.—Aspect of livers of Pair 1. N = Normal ; C = castrated partner. × 0·4.
Fig. 2.—Lung metastasis of hepatoma shown in Fig. 1. × 45.
Fig. 3.—Aspect of liver of Pair 9 *in situ*. N = Normal ; C = castrated partner.
Fig. 4.—Early adenocarcinoma found in seminal vesicle of normal partner (Pair 13). × 15.
Fig. 5.—Detail from Fig. 4. × 45.
Fig. 6.—More advanced carcinoma found in seminal vesicle of normal partner (Pair 11). × 25.
Fig. 7.—Another area of the tumour depicted in Fig. 6 showing destruction of muscular coat. × 45.
Fig. 8.—Detail from Fig. 7. × 300.
Fig. 9.—Area of tumour found in seminal vesicle (Pair 2) showing pronounced inflammatory changes and a giant cell near the centre. × 80.
Fig. 10.—Adenocarcinoma of the seminal vesicle. Tubular structures surrounded by coarse collagen fibres. × 80.
Fig. 11.—Anaplastic carcinoma of the seminal vesicle found in a " single " rat of the Sheffield strain treated with A.A.F. × 15.
Fig. 12.—Detail from Fig. 11. × 300.
Fig. 13.—Large nest of basophils found in pituitary of the intact partner (Pair 17). × 300.
Fig. 14.—Lesion found in kidney of the castrated partner of Pair 20. × 45.
Fig. 15.—Aspect of accessory sex organs, × 1·2.
Fig. 16.—Area of adenocarcinoma from nodule found in adhesion. × 45.
Fig. 17.—Metastasis attached to spermatic cord. × 25.

1

2

3

4

5

Bielschowsky and Hall.

Bielschowsky and Hall.

Bielschowsky and Hall.

by Doniach (1950). The same seems to hold good in the case of the seminal vesicle. The hyperplasia induced by increased amounts of androgens secreted by the stimulated testis enhances the susceptibility of the seminal vesicle to the carcinogenic action of A.A.F. The fact that tumours of macroscopic size were found only in rats treated with 24 or more doses of 4 mg. of A.A.F. and not in animals receiving less suggests that considerably more of the carcinogen is required to bring about tumour development in the stimulated seminal vesicle than was needed for the induction of adenomata in the hyperplastic thyroid (Hall, 1948).

That only one minute tumour of the seminal vesicle was found in an intact male joined to a spayed female is probably due to the lower dosage of A.A.F. given to this group and not to the nature of the gonadotropins of the spayed female. Only 17 to 20 doses of the carcinogen were administered because great difficulties were encountered in keeping alive pairs of opposite sex for periods necessary for tumour development. However, our material is not large enough to decide this question.

To summarize, we consider the tumours of the seminal vesicle to be due to a hormonal imbalance created by gonadotropins in the normal partner. These hormones, by stimulating the gonads of the normal partner, increase the androgen production of its testes and therefore are responsible for the hyperplasia of the seminal vesicles. Although unable to promote tumour growth in the testes they bring about conditions in which an accessory sex organ becomes susceptible to the action of A.A.F. The possibility cannot be excluded that still more prolonged androgenic stimulation may ultimately bring about tumour formation in the seminal vesicle without the aid of A.A.F.

ADDENDUM (received for publication February 7, 1951).

After the above paper had been submitted an additional pair of parabiotic rats had to be sacrificed. In this pair a normal male was joined in parabiosis to a castrate, the former having received 20 doses of A.A.F. by stomach tube. In the 36th week of the experiment the health of the normal partner declined rapidly. Besides an enlarged liver two hard nodules of approximately the size of a cherry could be palpated in the lower part of the abdomen. The post-mortem examination confirmed the presence of a greatly enlarged liver containing multiple benign cystic cholangiomata. The abdominal nodules were, in fact, the two seminal vesicles and coagulating glands which were found transformed into irregular masses (Fig. 15). They were white in colour, hard, and solid throughout. When cut, the lumen of the glands was not recognizable. Histological examination revealed that most of the normal tissue had been destroyed by an adenocarcinoma which was of similar structure to the smaller neoplasms of the seminal vesicle previously described. Adhesions connected the left seminal vesicle with loops of small intestine and a hard small nodule was found embedded in these adhesions at approximately 3 mm. distance from the upper margin of the left tumorous gland. Fig. 16 shows the microscopic appearance of the lesion. Histologically it consisted of acini lined by atypical epithelium surrounded by a fairly cellular connective tissue containing a few collagen fibres. Comparison with the neoplastic seminal vesicles showed close resemblance in structure, but it could not be decided whether the nodule represented a blood or lymph borne metastasis or whether this deposit of tumour tissue was the result of continued growth of the primary tumour into the loose tissue of the adhesion. However, a true metastasis was found at another site. Attached to the posterior and lateral surfaces of the left spermatic cord above the point where it was severed a small nodule was present, just recognizable in Fig. 15. It proved to be an area of adenocarcinoma which had invaded the outer muscular layer of the spermatic cord (Fig. 17). It had the same structure as the primary tumour and the nodule present in the adhesion.

This case proves that the neoplastic lesions found in seminal vesicles of normal male rats treated with A.A.F. and joined in parabiosis to a castrate can progress to the state of true malignancy.

### SUMMARY.

(1) The tumours induced by A.A.F. in normal male rats joined in parabiosis to castrated or spayed litter-mates have been described. Neoplasms were only obtained in animals receiving the carcinogen and never in their untreated litter-mates.

(2) Benign or malignant tumours of the liver were found depending on the amounts of the carcinogen given and on the duration of the experiment. The malignant hepatomas metastasized to the lungs of the animal bearing the cancer of the liver, but never " infected " the other partner.

(3) Adenocarcinomata of the seminal vesicles were found in 5 rats. These neoplasms are considered to be due to two factors ; the carcinogenic action of A.A.F. and the hyperplasia induced in the seminal vesicle by high levels of androgens.

A further example of adenocarcinoma of the seminal vesicle is described in an addendum. The tumour was larger than those previously encountered and had metastasized.

### REFERENCES.

Bielschowsky, F.—(1944) *Brit. J. exp. Path.*, **25**, 90.—(1945) *Ibid.*, **26**, 270.
Biskind, M. S., and Biskind, G. R.—(1944) *Proc. Soc. exp. Biol. N.Y.*, **55**, 176.—(1945) *Ibid.*, **59**, 4.
Doniach, J.—(1950) *Brit. J. Cancer*, **4**, 223.
Flexner, S., and Jobling, J. W.—(1907) *Zbl. allg. Path. path. Anat.*, **17**, 257.
Gee, E. M.—(1948) *Brit. J. Urol.*, **20**, 72.
Hall, W. H.—(1948) *Brit. J. Cancer*, **2**, 273.
Jacobsohn, D.—(1948) *Acta physiol. scand.*, **17**, suppl., 57.
Lazarus, J. A.—(1946) *J. Urol.*, **55**, 190.
Lipschutz, A.—(1950) 'Steroid Hormones and Tumours.' Baltimore (Williams & Wilkins Co.).
Martins, Th.—(1929) *C. R. Soc. Biol., Paris*, **103**, 1341.
McCree, L. E.—(1948) *J. Amer. med. Ass.*, **136**, 679.
Paschkis, K. E., Cantarow, A., and Stasney, J.—(1948) *Cancer Res.*, **8**, 257.
Selye, H.—(1947) 'Textbook of Endocrinology.' Univ. de Montreal (*Acta Endocrinologica*).
Strömbeck, J. P., and Ekman, B.—(1949) *Acta path. microbiol. scand.*, **26**, 480.
Twombly, G. H., Meisel, D., and Stout, A. P.—(1949) *Cancer*, **2**, 884.
Zahler, H.—(1950) *Virchows Arch.*, **317**, 547.
Zeckwer, I. T.—(1946) *Endocrinology*, **38**, 249.